# “It’s a secret between us”: a qualitative study on children and care-giver experiences of HIV disclosure in Kinshasa, Democratic Republic of Congo

**DOI:** 10.1186/s12889-021-10327-5

**Published:** 2021-02-06

**Authors:** Elysée Manziasi Sumbi, Emilie Venables, Rebecca Harrison, Mariana Garcia, Kleio Iakovidi, Gilles van Cutsem, Jean Lambert Chalachala

**Affiliations:** 1 Médecins Sans Frontières, Kinshasa, Democratic Republic of Congo; 2 Southern Africa Medical Unit, Médecins Sans Frontières, Main Road, Observatory, Cape Town, South Africa; 3 Division of Social and Behavioural Sciences, School of Public Health and Family Medicine, University of Cape Town, Cape Town, South Africa; 4 Carolina Population Center, University of North Carolina at Chapel Hill, Chapel Hill, NC USA

**Keywords:** Children, Democratic Republic of Congo, HIV care continuum, Disclosure, Qualitative research

## Abstract

**Background:**

It is estimated that 64,000 children under 15 years of age are living with HIV in the Democratic Republic of Congo (DRC). Non-disclosure – in which the child is not informed about their HIV status - is likely to be associated with poor outcomes during adolescence including increased risk of poor adherence and retention, and treatment failure. Disclosing a child’s HIV status to them can be a difficult process for care-givers and children, and in this qualitative study we explored child and care-giver experiences of the process of disclosing, including reasons for delay.

**Methods:**

A total of 22 in-depth interviews with care-givers and 11 in-depth interviews with HIV positive children whom they were caring for were conducted in one health-care facility in the capital city of Kinshasa. Care-givers were purposively sampled to include those who had disclosed to their children and those who had not. Care-givers included biological parents, grandmothers, siblings and community members and 86% of them were female. Interviews were conducted in French and Lingala. All interviews were translated and/or transcribed into French before being manually coded. Thematic analysis was conducted. Verbal informed consent/assent was taken from all interviewees.

**Results:**

At the time of interview, the mean age of children and care-givers was 17 (15–19) and 47 (21–70) years old, respectively. Many care-givers had lost family members due to HIV and several were HIV positive themselves. Reasons for non-disclosure included fear of stigmatisation; wanting to protect the child and not having enough knowledge about HIV or the status of the child to disclose. Several children had multiple care-givers, which also delayed disclosure, as responsibility for the child was shared. In addition, some care-givers were struggling to accept their own HIV status and did not want their child to blame them for their own positive status by disclosing to them.

**Conclusions:**

Child disclosure is a complex process for care-givers, health-care workers and the children themselves. Care-givers may require additional psycho-social support to manage disclosure. Involving multiple care-givers in the care of HIV positive children could offer additional support for disclosure.

**Supplementary Information:**

The online version contains supplementary material available at 10.1186/s12889-021-10327-5.

## Background

HIV prevalence in the Democratic Republic of Congo (DRC) is estimated to be 0.8% amongst adults aged 15–49. Approximately 450,000 people in DRC are living with HIV, with 57% of them receiving antiretroviral (ARV) treatment [1]. According to UNAIDS, of all adults aged 15 years and over living with HIV, 62% were on treatment, compared to only 25% of children aged 0–14 years [1]. In 2015 approximately 1.8 million children globally were living with HIV and 400 children were newly infected each day. Of these children living with HIV, an estimated 49% have access to the ARV treatment they require [2]. It is estimated that there are 64,000 children under the age of 15 living with HIV in DRC [1].

One of the challenges for children living with HIV in any context is the disclosure process during which they learn about their HIV status. Disclosure can happen at any time – some children may learn that they are HIV positive at the moment of testing, whereas others may not be told until they have been on ARV treatment for many years. Literature on adolescent HIV disclosure includes studies exploring how young people share their HIV status with others, such as sexual partners, friends or other relatives [3, 4], but for the purposes of this study, we use disclosure to mean telling a child who is not yet aware of their virological status that they are HIV positive. The process of disclosure is more than a single event and can happen in many different ways. In addition to the direct implications of HIV disclosure to individual children, care-givers and health-care workers are often confronted with challenges in managing the process and the reaction of the child.

The World Health Organisation (WHO) recommends that children of school-going age should be informed of their HIV status [5]. *Médecins Sans Frontières* (MSF) guidelines on child disclosure recommend that children under 12 should undergo a process of progressive disclosure, in which they are gradually informed about what is happening in their body, with HIV/AIDS finally being named at the end of the process [6]. These guidelines also recommend that this process should begin when the child understands the concepts of illness [6].

HIV disclosure to children at the *Centre Hospitalier de Kabinda* (CHK) in Kinshasa, DRC takes place in a stepwise manner; starting from around five years old until the child has been fully disclosed to. Full disclosure ideally happens before the age of 12. The health-care workers first discuss the disclosure process with care-givers, who are often the biological parents of the child. They assist them in disclosing to the child, or, with the consent of the care-giver, they disclose to the child themselves, over two partial disclosure sessions. During these partial disclosure sessions, children are informed about their body, health, immunity, the need to take medication and to attend the clinic regularly for medical consultations and blood tests. All this is explained using visual aids without mentioning terms such as HIV, AIDS, CD4 or ARVs. When children are considered ready to learn about their HIV status, a full disclosure session takes place, where all the information about health status, disease, treatment and modes of transmission is revealed to the child [6]. An additional challenge relating to disclosure in CHK and across DRC is that children can only be informed of their HIV status with the approval of their care-giver. This, along with the legal age of consent for HIV testing in DRC being 18, can cause delays in the process [7].

As described in the 2011 WHO guidelines on disclosure, there is evidence of the health benefits of disclosure including improved treatment adherence [8, 9], and little evidence of psychological or emotional harm from telling a child about their HIV status [5]. The role of care-givers in the process is very important, and there are several studies from different contexts in Sub-Saharan Africa discussing their involvement in disclosure, with Madiba’s study in particular looking at challenges associated with reasons for delaying the process [10–12]. A systematic review looking at disclosure to children has shown, however, that as well as benefits to disclosing to children, there may also be neutral or negative effects on their psychological well-being and adherence [13–15].

Barriers to disclosure include a fear of stigmatisation of the child and their wider family [16]. Other barriers include fearing creating conflict or concern that a care-giver is ‘robbing’ the child of their youth by burdening them with knowledge of their HIV status [10]. Additional challenges identified in the literature with regards to disclosing to young people include a lack of parent or guardian knowledge and feeling uncomfortable talking about issues surrounding sexuality [17].

We conducted this qualitative study to explore the experiences of HIV disclosure for children and their care-givers in Kinshasa, DRC, with a particular focus on the barriers to disclosure from the perspective of the care-givers. We explored why some care-givers had disclosed to their children and why others had not, as well as how children experienced the disclosure process. One of the unique aspects of this study was that it was conducted to directly inform programmatic activities in CHK, and to help MSF understand more about the process of disclosure in this particular context. The study seeks to contribute to literature on disclosure from an HIV programme within a Francophone African setting.

## Methods

### Study setting

This qualitative study was carried out in one health-care facility, CHK, in Kinshasa between July and August 2017. MSF has been providing HIV and TB care at CHK since 2002, including free in-patient and out-patient services.

### Sampling and recruitment

Purposive sampling was used to select 11 care-givers who had disclosed to their children, and 11 who had not, in addition to 11 HIV positive children aged 15–19. Only children who knew their HIV status were invited to participate. HIV positive children who knew their status were identified from the clinic register and their care-givers were contacted either telephonically or were approached in the waiting room by one of the research team after their routine clinic appointment and invited to take part. Potential participants were informed that the study was being conducted to help improve MSF’s disclosure activities. Study staff ensured that potential participants knew the study was separate from any clinical appointments and was unrelated to their treatment, or that of their child. Whilst nobody directly declined the invitation to participate, there were several eligible children and care-givers whom it was not possible to trace, and who did not attend interviews as scheduled.

### Data collection and analysis

In-depth interviews took place in a private room within CHK. They were conducted in Lingala, audio-recorded and transcribed and translated into French. Interviews were conducted by a trained female researcher with a qualification in psychology, and experience working with HIV positive children in CHK. She has previously conducted research in this context, and is fluent in both French and Lingala. Her training consisted of data collection skills including the principle of saturation, translation, informed consent and data analysis.

Interviews ranged in duration from 14 to 41 min. Interviews with children took an average of 17 min, and those with care-givers an average of 26 min. Interview guides for care-givers and children explored similar issues surrounding disclosure from the different perspectives of the groups involved. Interviews were carried out until saturation was reached, in which no new data was emerging from the interviews. Each interviewee was interviewed once, and no repeat interviews were conducted. Interviews were conducted individually, unless the child wanted the care-giver to be present during their interview. The in-depth interview guides were developed by the research team and were piloted in the clinic before data collection began to verify the translation into Lingala and the wording of the questions. Interview questions were open-ended to encourage discussion, and to allow interviewees the space to discuss issues which were important to them (see interview guides in Additional file 1). Notes were also taken during the interviews.

### Data analysis

Transcription and translation (from Lingala to French) of the audio-recordings took place simultaneously. Manual coding and thematic analysis [18] were conducted by two co-investigators, who reviewed the transcripts independently before discussing the codes, sub-codes and emerging themes together. Any discrepancies in coding (such as the two co-investigators attributing different codes or interpretations to the same interview extract) were clarified through discussion and to ensure investigator triangulation. Codes were based on a thorough reading and re-reading of the transcripts, in which the two co-investigators looked for relevant information in the direct words of the interviewees. The codes were grouped into three main themes which are presented in Table 1 below. Preliminary findings were discussed with the wider research team for validation before continuing. Thematic analysis was used to assist the investigators in the identification, analysis, organisation, description and reporting of themes [18].

**Table 1:** Summary of key themes, sub-themes and interviewee group

Codes and sub-codes	Emerging themes heme	Interviewee group
Experiences Planned or unplanned Accidental disclosure Place of disclosure Care-giver involvement in disclosure	Experiences of the disclosure process	Care-givers who had disclosed to their children, and children.
Reasons for disclosing (benefits) Overall benefits of disclosure Improved adherence Improved communication Improved health outcomes Increased independence of the child	Reasons for disclosure	Care-givers who had disclosed to their children. Children were asked to reflect upon the potential benefits of disclosure.
Reasons for not-disclosing (perceived negative effects) Shame and guilt Being HIV positive Fear of gossip Multiple care-givers being responsible for the child Fear of causing distress to the child Not having enough information to do so	Reasons for non-disclosure	Care-givers who had not disclosed to their children. Children and care-givers who had not disclosed were asked to reflect more broadly on why other people may not disclose.

### Ethical considerations

Verbal informed consent was taken from all care-givers and children ≥18 before interviews began. Verbal informed assent was taken from interviewees 15–18 years old, in addition to verbal informed consent from their care-giver. This study was approved by the *Médecins Sans Frontières* Ethics Review Board, Geneva, Switzerland (1724) and the Ethics Committee of the School of Public Health, University of Kinshasa, DRC (032/2017). Children who had not been disclosed to were not eligible for the study so as to respect the decision of their care-givers about not wishing to disclose to them. This was verified by the counselling team before data collection began. As the sample of HIV positive children and care-givers included in this study is small, only their age range rather than their actual age is included to prevent them from being identifiable.

## Results

A total of 22 care-givers and 11 children participated in this study. Of the care-givers, 11 had disclosed to their children and 11 had not. At the time of interview, the mean age of children and care-givers was 17 [15–19] and 47 (21–70) years old, respectively. Care-givers included biological parents, grandmothers, siblings and community members and 86% of them were female. Many care-givers had lost family members due to HIV.

The main themes presented below are: experiences of the disclosure process; reasons for disclosure and reasons for non-disclosure from the perspective of care-givers and children. They are also summarised in Table 1. Experiences of disclosure differed and included those who had found out accidentally prior to attending CHK, and those who had been disclosed to at a health-care facility. Perceived benefits of disclosure included improved adherence, better communication and improved health. Reasons for non-disclosure were complex, and included care-giver feelings of guilt or shame, fear that the child would talk about their HIV status with others and concerns about the child and wider family experiencing stigmatisation.

### Experiences of disclosure

Care-givers and their children had differing experiences of the disclosure process, including planned disclosure at a health-care facility, planned disclosure at home and unplanned disclosure. Some children had been disclosed to by health-care workers and others by family members.

Several children suspected they had an illness before they were disclosed to, asking why they had to take daily medication or were ‘*always ill’*. One girl (aged < 18) described how she asked her aunt why she had to take medication, after she had repeatedly asked her questions about her health:

I asked myself questions because I took medication every day, but I didn’t know why until the moment that they told me. They didn’t say anything to me [when I asked].

Some interviewees had experienced the process of disclosure ‘*little by little’* at CHK and were able to explain the counselling process in which images were used to explain the child’s HIV status to them. Several children were able to explain that HIV was a virus, and that it could be treated ‘*just like any other’*. This teenage boy describes how disclosure took place at a hospital when he was younger, when a counsellor had disclosed to him:

I came to the hospital with my mother and the test said that I was infected, so they prescribed me treatment to follow. I was six when they did the test but 12 when they told me. I was at the hospital and the counsellor told me. I was alone – I was the only one they called in and my mother stayed in the waiting room. They told me that ‘life continues with HIV’.

One mother, also HIV positive, with a teenage daughter described the distressing way her child had found out their HIV status prior to receiving care at CHK:

They tested her and she came back in tears with a paper in her hand. [The nurse] shouldn’t have told her like that. The result should be managed between the medical team and the mother of the child, who should be the one to disclose. And they knew that I was HIV positive too! I was very angry. I was scared for her when they told her: I was scared because I have the HIV virus, and I didn’t think it could happen to my child.

It was also argued by interviewees, such as in the above example, that it is better for a child to find out their HIV status through planned disclosure than to find out accidentally.

### Perceived benefits of disclosure

Care-givers and children perceived the benefits of disclosure to include improved adherence to antiretroviral treatment and an overall improvement in the child’s health, improved behaviour and communication between family members at home.

Care-givers believed that the child would become more autonomous, independent and responsible for their own health if they knew their HIV status, and that disclosure would help them to understand why they are taking treatment daily, and thus improve adherence:

I was scared when I told him; scared that he would worry. You should tell the truth to children so that they take their treatment: I’ve heard that lots of children die because their mothers hide the truth from them. He’s much better now. Before, he used to have a fever all the time but now he’s much better. He became nicer. Now, when he goes out he takes his treatment. If he’s not far from the house and if he notices it’s time to take his treatment, he comes home quickly to take it. If he’s going to another neighbourhood, he goes out with his medication and his bottle of water.

Mother of a teenage son

One boy (aged < 18) described how learning that he was HIV positive helped him to take his treatment more easily, and be in better health:

It’s not difficult [to take treatment]. It’s good because there’s been a change. When I let things go before I couldn’t play football, but since I’ve started to look after myself I am good at football. Basketball too. There’s a difference [since disclosure] because I take my treatment better so I can be in better health.

### Reasons for non-disclosure

Care-givers who had not disclosed to their children gave several reasons why they had not done so and explained their concerns about disclosure. These included wanting to protect themselves and other family members from potential stigmatisation and fearing that their children would tell other people about their HIV status if they were disclosed to because they ‘*talk too much’*. The phrase ‘*keep a secret*’ was frequently used in reference to HIV, and not wanting others to know the status of the child. Children did not reflect on potential reasons for non-disclosure as much as the care-givers, but this teenage boy talked about the stigmatisation he thought could occur when sharing one’s HIV status with someone else:

There are some things children with HIV can’t do. Like talk to people in the same way. And other people might think they can’t eat from the plates they use. And if other people learn that he has HIV he won’t have any friends, and no-one will touch his things. They’ll say that he has HIV.

Some of the care-givers wanted to ‘protect’ other family members from further distress and worry, so did not want to tell their children that they were HIV positive in case other family members found out in turn. This was particularly the case if the family had already lost other relatives to HIV. Several care-givers had also not told their families about their own HIV status, thus were reluctant to disclose to their children, or tell their child that they were also HIV positive because of the additional distress that may occur.

This HIV positive care-giver who was looking after her teenage son described her concerns and why she did not want him to learn about his HIV status:

When he was nine, they suggested that we should disclose his status to him but we refused because the child is very grown up. He talks too much. He’d tell his friends, even other adults or family members. It would become a subject of mockery. That’s why I refused. I decided that he couldn’t be told his status as he’d tell all of my family. Nobody in my family knows our situation. That’s why I don’t want to disclose.

Female care-giver

This grandmother, a primary care-giver, had not disclosed to her grandson because she wanted to stop him from ‘worrying’, and wanted to prevent other people finding out:

If he asks me questions, I say ‘you’re sick, no? Take your medication!’ It’s painful for someone to have to take medication every day and when I give them to him I do it in secret because I don’t want other people to find out. I tried to disclose to him, but I saw how worried he was and I stopped. I want to wait until he’s older … 15 or 16.

Grandmother caring for her grandson

HIV positive care-givers were also reluctant to disclose to children who had been perinatally infected because they felt guilty and did not want to be blamed by their children, or by other people for transmitting the virus.

Others wanted to protect their children from learning their HIV status in case it had negative consequences on their physical or mental health, and in several cases, interviewees did not believe the child was ‘*strong enough’* to learn the truth. There was a perception amongst care-givers and children that there could be a risk of negative thoughts and suicide relating to disclosure which prevented them from disclosing. One interviewee (aged < 18) thought that some care-givers did not disclose to their children as they believed the child may then refuse to take their treatment, resulting in ill health or death.

Two care-givers did not want to disclose to their children as they described them as ‘disabled’ (referring to cognitive impairment): one care-giver stated that there was ‘no point’ disclosing to her daughter as she would not understand the information given to her.

Another reason given by care-givers for non-disclosure was the fear that children may then ‘seek revenge’ by infecting other people with HIV, thus they did not tell their children about their status to prevent this from happening.

A further reason care-givers gave for non-disclosure was that not all the children in the study were biologically related to the care-givers responsible for them. Several of the adult interviewees were caring for a niece or nephew, grandchild or in one case, a child who was in their care through a community organisation. When describing their daily routines, children talked about staying with different relatives (often aunts) and moving between households, particularly at mealtimes. In many cases the movement between households was due to the death of the child’s primary care-giver from HIV-related illness and there was then a need to support the child through an extended family network. In some cases, the non-primary care-giver being unaware that the child was HIV positive or not having ‘permission’ from the primary care-giver (who could be the biological parent) to disclose caused delays in the process of disclosure.

This woman was one of several care-givers for her < 18 year old sister:

She takes her treatment well if it’s me and not someone else. If it’s someone else, she sulks, but if it’s me she doesn’t refuse it and she takes it properly. Sometimes I have other things to do so I have to leave the house, and if it’s the time for her to take her treatment, other people give it to her without knowing why. I told father that it’s a [problem with her lungs] and that’s why she needs to take treatment.

Having multiple care-givers made managing treatment and disclosure complex as there was a shared responsibility for the child, and not all care-givers were involved in supporting and providing for the children in the same way or at the same time.

## Discussion

This study contributes to existing literature from sub-Saharan Africa by describing the experiences of disclosure to children about their own HIV status from the perspective of care-givers and children themselves. We have described the reasons for disclosure and non-disclosure from both perspectives, as well as exploring experiences of the disclosure process. We have also considered the role of multiple care-givers in providing support to HIV positive children during disclosure. We demonstrate that disclosure is a complex process, and that care-givers and children experience it in different ways and have differing views on whether or not disclosure should happen. We show that some care-givers do not want to disclose to their children because of their own guilt and concerns about causing distress to the child.

Differing perspectives exist about the effects of disclosure, and experiences of this process from both perspectives can assist in improving disclosure programmes and supporting HIV positive children in the future. The benefits of disclosure, particularly the belief that disclosure can improve adherence; improve communication between care-givers and their children; improve the child’s health and enable them to protect future sexual partners from HIV transmission by preventing them from unknowingly transmitting the virus to others when they reach adolescence, have been noted elsewhere [19–22]. It is, however, important to note the documented potential negative or neutral effects of disclosure upon adherence that have been observed in studies from other contexts [4, 13–15]. These are summarised in a review by Nichols et al. which describes ‘conflicting results regarding the impact of disclosure on adherence’, with five studies showing a positive impact, five showing no association and four showing no association between disclosure and adherence [23].

Literature from the sub-Saharan Africa region highlights the difficulties that care-givers may face when thinking about disclosing to their children and the assistance that may be required from health-care workers [10, 24–26]. There are several studies on disclosure which discuss how guilt or fear may prevent care-givers from disclosing to their children in the case of perinatal infection, which was also the case amongst our interviewees [10, 16]. Our results point to the need to address the fears, shame and guilt of care-givers in order to strengthen their own process of acceptance of their own HIV status and that of their child’s, and support and assist with self-management of the disease. In addition, many care-givers are likely to have lost other partners or family members to HIV, thus may also need support to cope with their own grief.

As Vaz et al. have shown in a similar study from DRC, care-givers would welcome assistance and support from health-care workers to disclose to their children [21, 22]. We suggest that the disclosure process should be carried out in a supportive and structured way, creating a safe space for children and adolescents with HIV to grow, and for their care-givers to also get the support they need during and after the process. Offering ongoing support is essential for both children and care-givers and support cannot stop after ‘naming’ HIV, regardless of how the child became aware of the virus [26].

Another reason care-givers did not want to disclose to the children was the fear of the child and the family being stigmatised if the child tells other people that they are HIV positive. Previous research undertaken in CHK has shown the significance of HIV-related stigma [27] and how this can affect disclosure to others, affect adherence and the physical and mental health of the individual. Stigmatisation can prevent people from seeking support and health-care, and prevents care-givers from disclosing to other family members who could support them. Whilst group sessions for HIV positive youth and their care-givers currently exist in CHK, the potential for stigmatisation is important to consider when implementing such support structures.

Many studies from sub-Saharan Africa have found that cultural norms meant care-givers did not talk about sexuality or HIV prevention with their children, also contributing to preventing the child from learning about their HIV status [28–30]. Whilst this was not a direct finding in our study, it may be worth considering this in the ongoing child disclosure programme in CHK as well as in similar programmes elsewhere. This is particularly important to consider if the care-giver is an older relative, such as a grandmother of an HIV positive child.

The results of our study also suggest that it is important to consider who the care-givers of each child are when offering counselling and psycho-social support, as many young people have multiple care-givers and information about HIV disclosure and treatment may not flow between them. Ensuring that care-givers are asked if there is more than one person responsible for looking after the child (including at weekends, during school holidays or if the primary care-giver is working) would help to identify who is involved in giving support to the child. Such support includes helping children to take their medication by reminding them, giving it to them directly or supervising them, providing food to eat with the medication and offering emotional support. In DRC it is common for children to belong to multiple households and be cared for by different people, particularly for those who have lost relatives to HIV, and this needs to be reflected in the provision of services by ensuring that non-primary care-givers are identified and also given the support they may need through formal counselling, support groups, or more informal support.

Our study findings support the relevance of activities which MSF has already implemented at CHK to further engage with care-givers. Staff continue to make home visits and phone calls to identify and engage with care-givers who are difficult to locate. If the care-giver accompanies the child to their clinic appointment, staff engage them in discussion about the benefits of disclosure, offer them psycho-social support and help to identify the barriers preventing them from wanting to disclose to their child. Such approaches support the previously-cited literature on the role of health-care workers, particularly counsellors, in the disclosure process, and how their involvement is often appreciated by care-givers who feel unable to manage the process themselves.

One limitation of our study is social desirability bias, as interviewees may have been reluctant to explain why they did not disclose to their children for fear of being judged. As this is a qualitative study, we were not able to look at the clinical outcomes of disclosure and as pointed out in the aforementioned systematic review, longitudinal, prospective research is needed to evaluate adherence over time. Our study only includes older children, thus the views of younger children were not included and due to the challenges of recruiting such a vulnerable and hard-to-reach population our sample size was limited. A final limitation is that we did not explore the perceptions of health-care workers, but felt that their views had been considered elsewhere in the literature.

## Conclusions

Finding the right time and way to disclose to a child is essential to reduce possible negative consequences for the child and care-giver. There is a need to plan and facilitate the disclosure process, including before and after telling the child that they are HIV positive to prevent accidental or distressing disclosure. Disclosure programmes need to consider involving and supporting secondary care-givers who are also involved in clinical follow-up and adherence support. Ongoing psycho-social support is also required for care-givers, particularly those living with HIV or who are grieving for relatives who died of HIV-related illness, to enable them to understand and cope with their child’s HIV diagnosis and assist with the process of disclosing to their child.

## Supplementary Information

**Additional file 1.**

## Data Availability

The datasets generated during and/or analysed during the current study are not publicly available due to the sensitive nature of the data collected about HIV status, but are available from the corresponding author on reasonable request.

## Abbreviations

ARV: Antiretroviral
AIDS: Acquired Immunodeficiency Syndrome
CHK: *Centre Hospitalier de Kabinda*
DRC: Democratic Republic of Congo
HIV: Human Immunodeficiency Virus
MSF: *Médecins Sans Frontières*
WHO: World Health Organisation
