# Supplementary material for: “It’s a secret between us”: a qualitative study on children and care-giver experiences of HIV disclosure in Kinshasa, Democratic Republic of Congo
Source: BMC Public Health. 2021 Feb 6;21:313. doi: 10.1186/s12889-021-10327-5 (PMC7866707; doi:10.1186/s12889-021-10327-5)
Supplement: Supplementary file 1 — Additional file 1. [file 12889_2021_10327_MOESM1_ESM.docx]

**In-depth interview guide: care-givers who have disclosed to their child**

Thank you for giving your informed consent and agreeing to talk to us today. You do not have to answer any questions you do not want to answer, and you are free to stop or leave the interview at any time. Do you have any questions for me before we begin?

Age of child caring for:

Age of parent/guardian:

Relationship with child:

1. Can you explain to me your relationship with your child?

**Probe:** biological child, caring for child for another relative, circumstances leading to caring for child such as death of relative, living arrangements

2. When did you find out that your child was HIV positive? Can you tell me how *you* found out?

**Probe:** sickness of child, who disclosed to them, suspicion of HIV, if child was on treatment before learning about HIV status, death of child’s HIV positive biological parent

3. How did you feel when you were told that your child was HIV positive?

4. Can you tell me how your child found out that they were HIV positive?

**Probe:** who told him/her, when, circumstances of disclosure, whether or not they were present

5. Can you explain to me exactly what happened when your child was disclosed to? How do you think their disclosure was conducted?

**Probe:** partial/full disclosure, who assisted with disclosure, accidental or planned disclosure

6. How did your child react when they found out they were HIV positive?

**Probe:** feelings of child, belief, understanding of HIV status, questions asked, questions asked about medication

7. Do you think anything has changed in your child’s life since they learned their HIV status? Can you give me any examples?

**Probes:** changes in behaviour, change in openness to talk about HIV

8. Do you feel your relationship with your child changed after they learned about their HIV status? Can you explain to me how? Do you have any examples you can give me?

**Probe:** changes in communication,

9. Have your relationships with other family members changed since learning about your child’s HIV status?

**Probe:** more or less support, disputes within the family, misunderstandings, stigmatisation, rejection

10. I’d like to ask you some questions about the treatment your child takes. Can you explain to me what you know about their treatment?

11. Can you tell me how well you think your child adheres to their treatment?

**Probes:** strategies for adherence, communication about adherence

12. Sometimes it is difficult to help children take their treatment. Can you tell me about any of the challenges you have faced, and how you have overcome them?

**Probe:** not understanding treatment, nobody to ask for help, working and not being able to supervise child, not being able to explain to child

13. Do you ever feel that you would like more support? What kind of support do you think you could benefit from?

14. What, in your opinion, are the benefits and the challenges with your child knowing their HIV status? Can you give me examples?

15. Is there anything that you think could have been done differently during disclosure, to help your child understand that they are HIV positive?

16. Sometimes care-givers are scared of disclosing to their children: what kind of advice would you give to people in this situation?

Thank you for your time today.

Do you have anything you would like to add, or any questions you would like to ask us?

**In-depth interview guide: care-givers who have not disclosed to their child**

Thank you for giving your informed consent and agreeing to talk to us today. You do not have to answer any questions you do not want to answer, and you are free to stop or leave the interview at any time. Do you have any questions for me before we begin?

Age of child caring for:

Age of parent/guardian:

Relationship with child:

1. Can you explain to me your relationship with your child?

**Probe:** biological child, caring for child for another relative, circumstances leading to caring for child such as death of relative, living arrangements

2. Can you tell me what you understand about HIV?

3. When did you find out that your child was HIV positive? Can you tell me how you found out?

**Probe:** sickness of child, who disclosed to them, suspicion of HIV, if child was on treatment before learning about HIV status, death of biological HIV positive relative

4. How did you feel when you learned that your child was HIV positive?

5. Has your child ever asked you any questions about their health? if yes, what kind of questions have they asked? What do you tell them when they ask?

6. Sometimes children who don’t know their HIV status ask why they have to take medication every day. Is this something you have experienced, and if so, can you tell me more about it?

7. Does your child take their treatment every day? Can you explain to me how they take it, and if there is anyone who helps/reminds them?

8. What does your child understand about the treatment they take? What do they think about it?

9. We know that it is often difficult for care-givers to tell their children that they are HIV positive. Have you also felt this way?

10. If yes, what kind of difficulties have you had in telling your child that they are HIV positive?

11. What, in your opinion, are the benefits and the challenges with your child knowing their HIV status? Can you give me examples?

12. Do you think that your child would be stigmatised if s/he found out? Why/why not?

13. If you were to disclose to your child, would you need any support? Can you tell us what would help you?

14. Is there anything we can do to help you through the process?

Thank you for your time today.

Do you have anything you would like to add, or any questions you would like to ask us?

**In-depth interview guide: care-givers who have not disclosed to their child**

Thank you for giving your informed consent and agreeing to talk to us today. You do not have to answer any questions you do not want to answer, and you are free to stop or leave the interview at any time. Do you have any questions for me before we begin?

Age of child :

Sex of child :

1. Can you tell me what a typical day is like for you?

**Probes:** school, work, friends, housework

2. Can you tell me what like is like at home?

**Probes:** where they live, who they live with, movement between households, relationships with family members

3. What do you understand by HIV?

4. How did you find out about your HIV status?

**Probes:** when, by who, presence of guardian or health-care worker, circumstances of disclosure

5. Before you learned about your HIV status, did you ever ask any questions about your health? What kinds of questions did you ask, and what kind of answers did you get?

6. Is it difficult for you to take medication every day? How do you find the experience?

**Probes:** school, friends, stigmatisation, adherence strategies

7. Since you found out your status, has the way you take your medication changed? Can you tell me about any of these changes?

8. What do you think are the biggest challenges for young people like you who are living with HIV?

9. Often, young people can find it difficult to accept their HIV status. When do you think young people should be disclosed to, and why?

10. In your opinion, what are the best circumstances for a young person to be disclosed to?

**Probes:** location, alone or accompanied, age

11. Sometimes care-givers find it difficult to tell their children that they are HIV positive. What do you think their reasons might be?

12. What do you think we can do to help young people who are living with HIV?

**Probes:** counselling, disclosure support, youth groups

Thank you for your time today.

Do you have anything you would like to add, or any questions you would like to ask us?
